# Invasive *Klebsiella pneumoniae* liver abscess syndrome complicated by carbapenem-resistant *Acinetobacter baumannii* infection: a case report

**DOI:** 10.3389/fmed.2024.1511734

**Published:** 2025-01-07

**Authors:** Qiupeng Feng, Hua Yuan, Jin Ma, Zhiqiang Guo, Xiaohua Xia, Guang Zhao

**Affiliations:** ^1^Department of Emergency Medicine, The First People’s Hospital of Kunshan, Kunshan, China; ^2^Jiangsu University Health Science Center, Kunshan, China

**Keywords:** *Klebsiella pneumoniae*, *Acinetobacter baumannii*, liver abscess invasion syndrome, therapy, case report

## Abstract

**Background:**

A liver abscess caused by hypervirulent *Klebsiella pneumoniae* can lead to multiple invasive extrahepatic infections, including lung abscesses, endophthalmitis, brain abscesses, and necrotizing fasciitis. This condition, known as *Klebsiella pneumoniae* liver abscess invasion syndrome, progresses rapidly and is associated with severe illness, high disability rates, and significant mortality. However, bloodstream infections with co-infection involving carbapenem-resistant *Acinetobacter baumannii* are exceedingly rare.

**Case presentation:**

The Emergency Medicine Department of the First People’s Hospital of Kunshan successfully treated a male patient diagnosed with liver, lung, and prostate abscesses. The patient underwent puncture and drainage, with analysis of the drainage fluid, sputum culture, and metagenomic next-generation sequencing (m-NGS) revealing a co-infection with blood-borne *Klebsiella pneumoniae* and *Acinetobacter baumannii*. Guided by drug sensitivity test results, the patient received treatment with polymyxin and cefoperazone sodium-sulbactam sodium for infection control and liver protection. The treatment was successful, and the patient fully recovered and was discharged.

**Conclusion:**

By reporting this rare case and highlighting the drug resistance of the bacteria, we propose a new diagnosis and treatment plan for managing *Klebsiella pneumoniae* combined with carbapenem-resistant *Acinetobacter baumannii* infection, along with a literature review.

## Introduction

*Klebsiella pneumoniae* (*KP*) is an anaerobic gram-negative bacillus that commonly inhabits the digestive, respiratory, urinary, and reproductive tracts of humans and animals. It can be classified into two types based on virulence and pathogenicity: *classic Klebsiella pneumoniae* (*cKP*) and *hypermucoviscous Klebsiella pneumoniae* (*hvKP*). *hvKP* carries genes regulating mucin-like substances and toxins, such as aerobactin, which are frequently linked to invasive diseases like liver abscess, intracerebral abscess, pulmonary abscess, endophthalmitis, and necrotizing fasciitis. These infections have high clinical mortality rates, posing a serious risk to patient health (1). In recent years, invasive syndromes caused by *KP* have garnered more attention in the medical community. However, cases involving simultaneous co-infection of *KP* and *Acinetobacter baumannii* (*AB*) in the same patient are extremely rare. This report presents the successful treatment of a patient at the Emergency Department of the First People’s Hospital of Kunshan, who suffered from a *KP* liver abscess invasive syndrome, complicated by an *AB* infection.

## Case presentation

The patient, a 39-year-old male, was admitted to the Emergency Intensive Care Unit (EICU) of Kunshan City First People’s Hospital, presenting with “fever and fatigue for 2 weeks.” He reported a long-standing history of diabetes, for which he had been taking oral medication to manage his blood sugar, though without regular monitoring or documentation of his blood glucose levels.

The patient developed a fever 2 weeks ago, with a peak temperature of 38.8°C. He also experienced occasional abdominal pain, particularly in the upper and middle abdomen. He did not have chills but reported occasional cough with sputum that was brick red and difficult to expectorate. The patient was treated with anti-infection (meropenem, moxifloxacin), liver protection, blood glucose control, and nutritional support. Abdominal enhanced CT revealed an abscess in the right lobe of the liver. Consequently, ultrasound-guided percutaneous puncture and drainage of the liver abscess were performed. The liver pus culture grew *KP*, while the sputum culture revealed *AB*. The patient’s treatment with a combination of meropenem and moxifloxacin was continued. The patient’s self-conscious symptoms were not improved, and he was transferred to our hospital for treatment.

**Figure 1:**
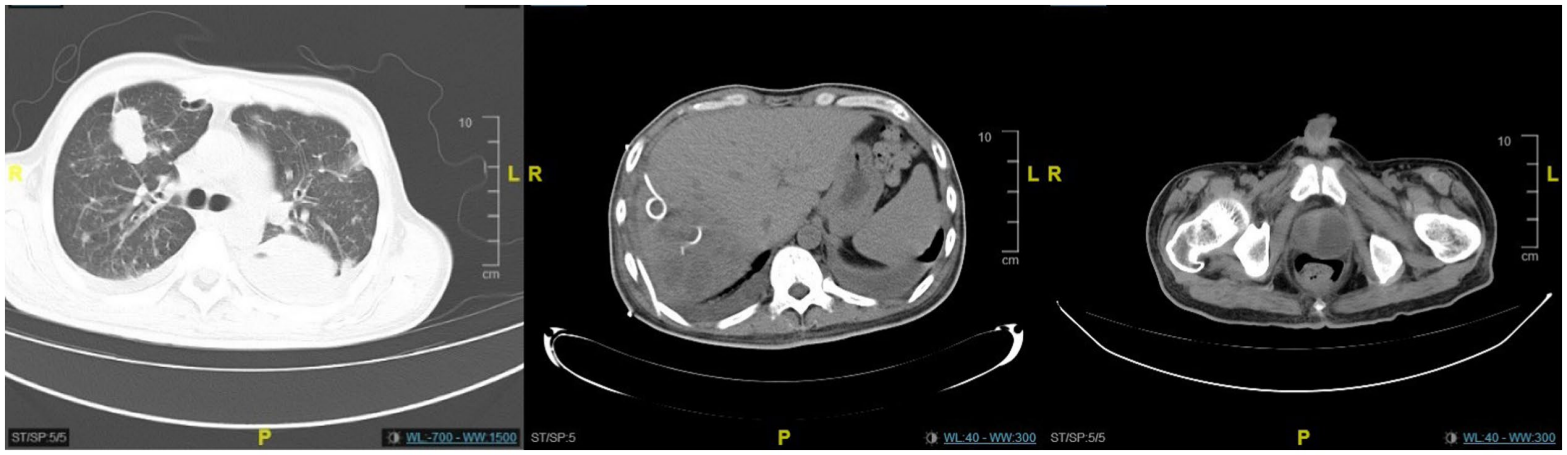
The CT scan revealed an abscess in the right lobe of the liver with multiple areas of pneumocephalus, an enlarged and distended prostate containing low-density lesions, and pelvic effusion. Bronchiectasis with infection was observed in the right lower lobe of the lung. Bilateral pleural effusion was present, along with distension of the lower lobes in both lungs.

The clinical and laboratory results from the emergency examination are detailed in Supplementary Table S1. CT imaging revealed progression of the lung lesions (Figure 1). Due to the patient’s worsening inflammatory markers, declining oxygen partial pressure and oxygenation index, and overall critical condition, he was transferred to the EICU for intensive care. Physical examination: T: 37.6°C, P: 133 beats/min, BP: 130/68 mmHg. He was conscious, emaciated, and seated, with tachypnea and coarse breath sounds in both lungs, accompanied by moist rales. The abdomen was soft, with tenderness in the right upper quadrant but no rebound tenderness. Bowel sounds were normal, and there was no edema in the legs. Subsequently, liver pus and sputum cultures revealed the growth of *AB*. Metagenomic next-generation sequencing (mNGS) of blood and sputum identified infections with both *KP* and *AB* (Supplementary Table S2). PET-CT examination showed combined lung infections with lung abscess formation and a large low-density lesion in the right lobe of the liver following liver abscess drainage; the prostate was found to be enlarged with decreased density, suggesting inflammatory changes (Supplementary Figure S1).

**Figure 2:**
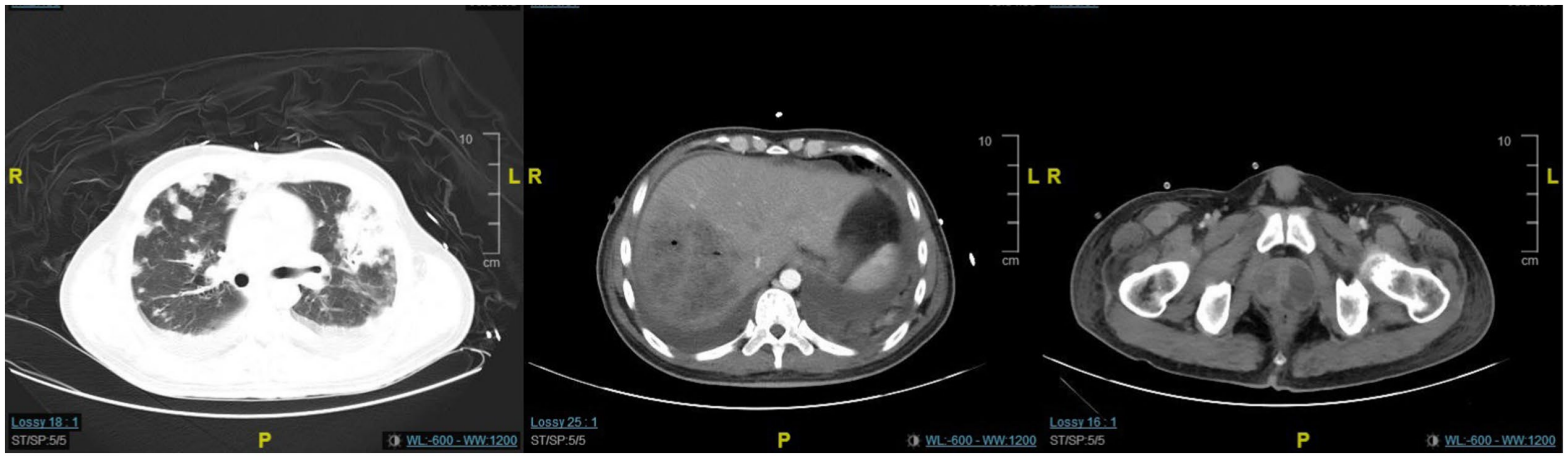
The CT scan revealed minimal improvement in the lung and prostate lesions, while the liver abscess showed significant resolution.

**Figure 3:**
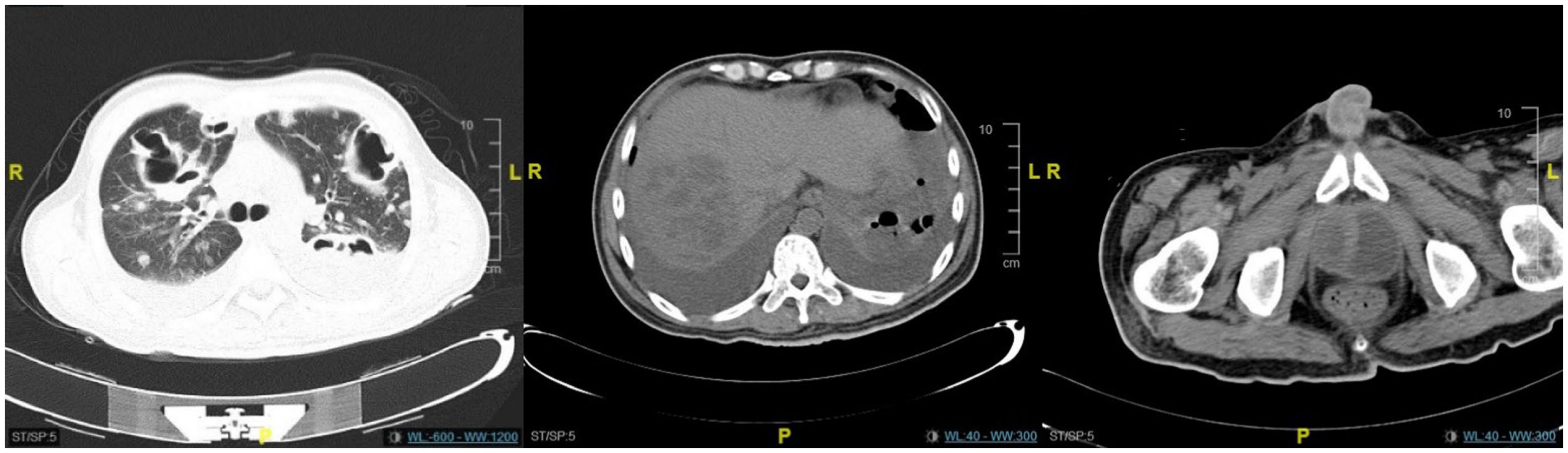
The CT scan revealed no significant improvement in the lung lesions, liver abscess, or prostate lesions.

**Figure 4:**
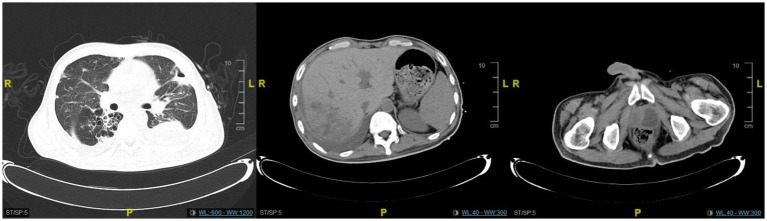
The CT scan revealed significant resolution of the lung and liver abscesses, while the prostate abscess showed only slight improvement.

The patient was given non-invasive ventilator-assisted ventilation, cefoperazone sodium-sulbactam sodium combined with polymyxin B for anti-infection, along with liver protection, anti-inflammatory treatment, and nutritional support. Considering the patient’s prostatitis and persistent infection, a perineal prostate puncture drainage was performed, with prostatic pus culture revealing *KP*. After 10 days of antimicrobial therapy, follow-up CT showed minimal improvement in the lung and prostate lesions but some progress in the liver abscess (Figure 2). Sputum and liver cultures continued to show *KP* and *AB* growth (Supplementary Table S3). Due to limited symptomatic improvement, the antibiotic regimen was adjusted to “cefoperazone sodium-sulbactam sodium combined with levofloxacin administered via intravenous drip, and amikacin via aerosol inhalation.” Five days later, a CT scan showed improvement in both the lung lesion and liver abscess, though the prostate lesion remained unchanged (Figure 3). Inflammatory markers had also improved compared to previous results. After 20 days, the patient underwent percutaneous drainage of both the lung and prostate abscesses. Cultures from the prostate pus and blood were negative. Cefoperazone-sulbactam sodium was gradually discontinued, and 2 weeks later, inflammatory markers showed significant improvement (Supplementary Table S1). The patient also discontinued amikacin aerosol inhalation. A follow-up CT scan 1 month later revealed marked improvement in both the lung lesion and liver abscess, with some improvement in the prostate abscess (Figure 4). The patient was discharged and, during a one-year follow-up, reported being able to perform daily activities. A chest CT confirmed significant improvement in the lung lesion (Supplementary Figure S2).

## Discussion

Studies have indicated that the colonization rate of *KP* in the intestines of the Chinese population reaches up to 75%, with approximately 22.8% identified as *hvKP* (2, 3). Liver abscesses caused by *hvKP* can lead to extrahepatic invasive infections, collectively referred to as invasive *Klebsiella pneumoniae* liver abscess syndrome (IKLAS). The male-to-female ratio for IKLAS is 1.5–2.5:1, and it is more prevalent in patients with diabetes. The mortality rate is higher than that of *cKP* infections, including those caused by multidrug-resistant strains, positioning it as a global public health concern (4).

Although IKLAS has garnered increasing attention, there remains a lack of optimal methods for identifying *hvKP*. In this case, m-NGS of sputum, pus, and blood detected *KP*. Based on these findings and the patient’s clinical progression, the infection was determined to be caused by *hvKP*. Diabetes mellitus or impaired glucose tolerance is a key risk factor for IKLAS. Studies have shown that factors such as diabetes, multiple abscesses, air-filled spaces within abscesses, metastatic infections, and septic shock are significantly associated with increased mortality (5). Hyperglycemia also provides a nutrient-rich environment conducive to bacterial growth and replication. Additionally, microcirculatory disturbances, vascular dysfunction, and structural damage in diabetic patients lead to hemodynamic changes, reduced chemotaxis of polymorphonuclear cells, impaired vascular endothelial cell migration, and diminished superoxide production (6). These factors are believed to contribute to the higher infection rates observed in diabetic individuals. In this patient, we believe that his long-standing diabetes, combined with years of poor glycemic control, played a significant role in the development of his infection.

In addition to its natural resistance to ampicillin, *hvKP* generally remains susceptible to multiple antibiotics. However, recent reports have highlighted the emergence of strains exhibiting both hypervirulence and drug resistance (7, 8). Although the *Klebsiella pneumoniae* in this patient did not show drug resistance, the co-infection with carbapenem-resistant *Acinetobacter baumannii* (CRAB) added a layer of complexity to the case (Supplementary Table S3). Co-infection between *KP* and *AB* is rare, but studies indicate that approximately 25% of patients with bloodstream infections present with polymicrobial infections (9). The synergy between *AB* and *KP* arises through mechanisms such as mutual protection, biofilm formation, and cross-feeding, which can enhance virulence and complicate treatment, potentially leading to worse clinical outcomes (10). In documented cases of such co-infections, poor outcomes have been observed. For example, a patient who developed postoperative incision infections after gastrointestinal surgery had both colistin- and tigecycline-resistant *KP* and *AB*, leading to the patient’s death; another case involved a patient with a pulmonary infection following cerebral hemorrhage surgery, complicated by co-infection with carbapenem-resistant *AB* and *KP*, resulting in a poor prognosis (11, 12). This case represents one of the rare instances of co-infection between *KP* and *AB*, further complicating treatment and underscoring the clinical challenges associated with such polymicrobial infections.

Treating complex cases such as this one is extremely challenging, with the first priority being the management of the primary abscess source. In this case, minimally invasive percutaneous puncture drainage was performed for both the liver and lung abscesses, resulting in positive outcomes. Although the prostate abscess did not fully resolve during the treatment course, the final culture of prostate pus showed no bacterial growth, and the patient experienced no significant symptoms. A year of follow-up revealed no need for additional surgical interventions, and no re-infection was observed.

The treatment of carbapenem-resistant gram-negative bacilli (CR-GNB) infections presents significant challenges. Selecting the appropriate antibiotics and devising a suitable treatment strategy are crucial for improving clinical outcomes and minimizing adverse reactions. In this case, we employed a combination of polymyxin and sulbactam. Studies have shown that high-dose sulbactam combined with another antimicrobial agent significantly enhances the rates of clinical improvement and cure when compared to other regimens (13). Nebulized inhalation therapy is a key component in maximizing local drug concentrations at the site of infection, particularly in respiratory infections. This approach allows the medication to effectively penetrate biofilms, such as those formed in the trachea, while minimizing systemic drug exposure and reducing toxicity. Nebulization of colistin and aminoglycosides is widely used for hospital-acquired pneumonia (HAP) and ventilator-associated pneumonia (VAP) caused by *Acinetobacter baumannii*. In this case, nebulization with amikacin sulfate was employed in the later stages and achieved favorable results, highlighting the efficacy of this targeted local treatment.

## Conclusion

This is a rare case of co-infection with *KP* and *AB*, in which the patient ultimately responded well to treatment. We believe the positive outcome is largely due to the absence of drug resistance in *KP*. The rise of multidrug resistance and polymicrobial infections has garnered significant concern among clinicians. The emergence of strains resistant to last-resort antibiotics, such as polymyxin and tigecycline, raises serious concerns about the potential onset of a “post-antibiotic era.”

## Data Availability

The raw data supporting the conclusions of this article will be made available by the authors, without undue reservation.
