# Supplementary material for: Invasive Klebsiella pneumoniae liver abscess syndrome complicated by carbapenem-resistant Acinetobacter baumannii infection: a case report
Source: Front Med (Lausanne). 2025 Jan 7;11:1511734. doi: 10.3389/fmed.2024.1511734 (PMC11753218; doi:10.3389/fmed.2024.1511734)
Supplement: Supplementary file 1 [file Supplementary_file_1.docx]

Supplementary Material

# Supplementary Figures and Tables

For more information on Supplementary Material and for details on the different file types accepted, please see [here](https://www.frontiersin.org/guidelines/author-guidelines#supplementary-material).

## Supplementary Table 1

| Laboratory variable | Admission | Discharge | Normal Value |
| --- | --- | --- | --- |
| WBC (10^9^/L) | 21.2 | 8.9 | 3.5-9.5 |
| N (%) | 95.2 | 77.7 | 40-75 |
| PLT (10^9^/L) | 130 | 198 | 125-350 |
| PCT (ng/mL) | 1.73 | 0.14 | 0.072-0.094 |

**Table 1:** Clinical and Laboratory variables

**Table 2:** Report on mNGS detection of pathogens

| **Genus** | | | | **Species** | | |
| --- | --- | --- | --- | --- | --- | --- |
| Type | Name | Relative abundance | Sequence number | Type | Confidence | Sequence number |
| Sputum | | | | | | |
| G^-^ | *Acinetobacter* | 14.8% | 66,472 | *Acinetobacter baumannii* | 99% | 25,573 |
| G^-^ | *Klebsiella* | 0.4% | 2,188 | *Klebsiella pneumoniae* | 99% | 568 |
| Blood | | | | | | |
| G^-^ | *Klebsiella* | 55.5% | 689 | *Klebsiella pneumoniae* | 99% | 146 |
| G^-^ | *Acinetobacter* | 6.5% | 64 | *Acinetobacter baumannii* | 99% | 20 |
| G^+^ | *Enterococcus* | 0.6% | 4 | *Enterococcus faecium* | 99% | 4 |

**Table 3:** Results of drug susceptibility tests

| ***Klebsiella pneumoniae*** | **MIC** | ***Acinetobacter baumannii*** | **MIC** |
| --- | --- | --- | --- |
| Ticarcillin | ≥128 | Meropenem | ≥16 |
| Cefoperazone sodium-sulbactam sodium | ≤8 | Imipenem | ≥16 |
| Levofloxacin | ≤0.125 | Polymyxin | ≤0.5 |
| Amikacin | ≤2 | Tigecycline | 2 |

## Supplementary Figure 1


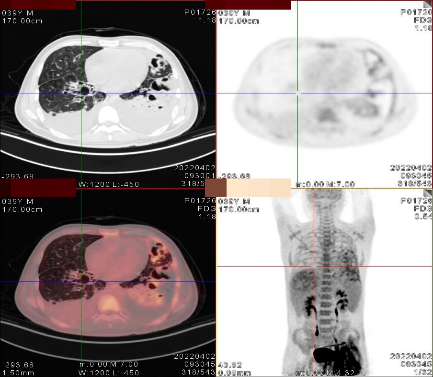

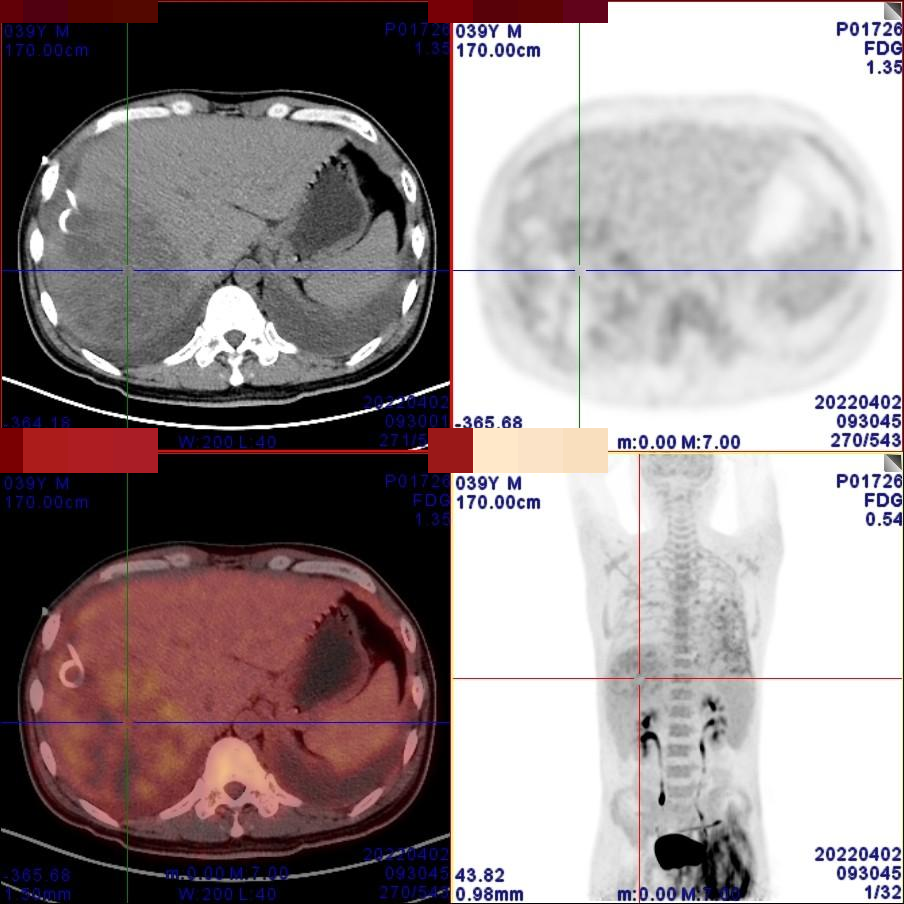

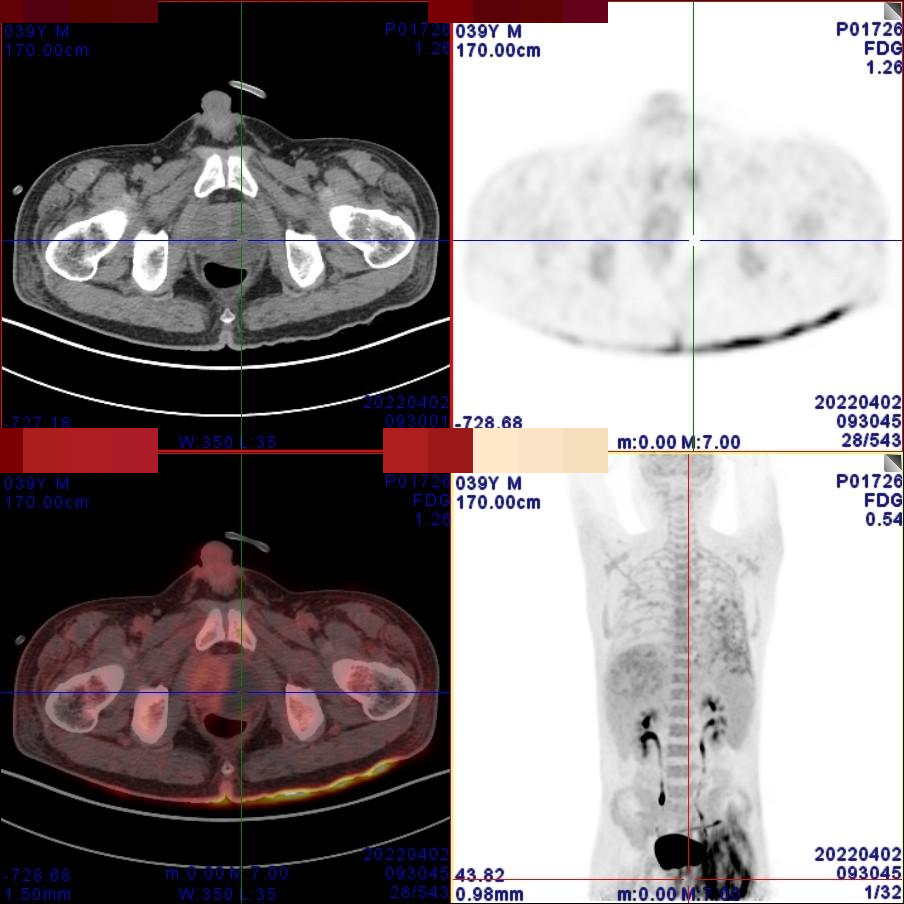


The PET-CT revealed bilateral lung infection with multiple areas of consolidation, lung abscess formation, partial atelectasis in the lower lobes, bilateral pleural thickening, and pleural effusion. Following liver abscess drainage, a large low-density lesion in the right lobe of the liver with increased glucose metabolism and subcapsular effusion was observed. Additionally, the prostate was found to be enlarged, with decreased density and heterogeneously increased glucose metabolism in the right peripheral zone, suggesting inflammatory changes.

**Supplementary Figure 2**


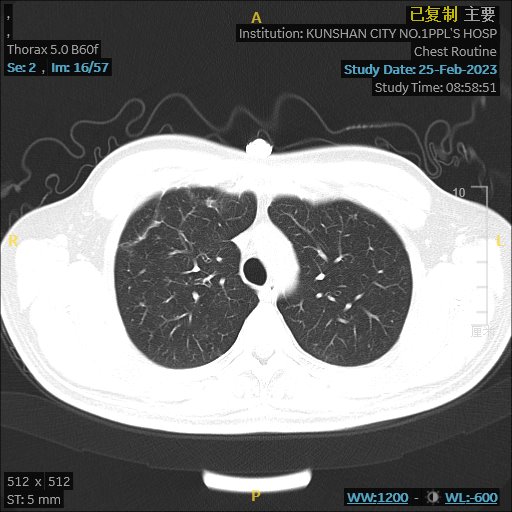

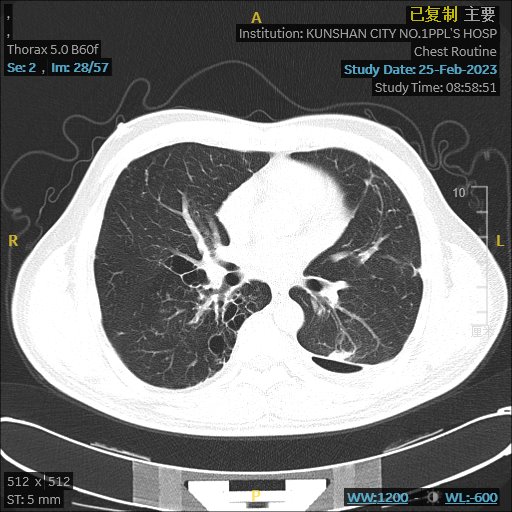

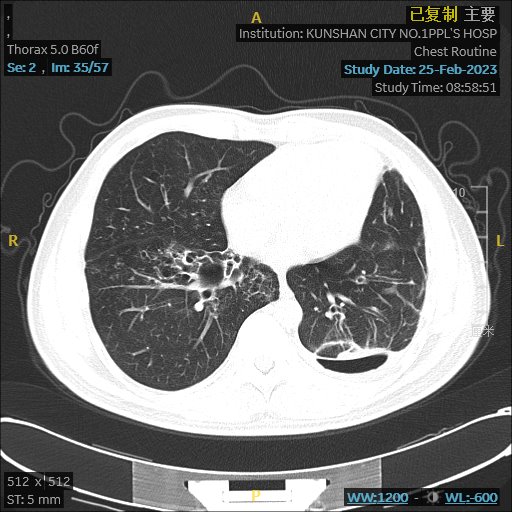


The CT scan revealed significant absorption of the lung lesions.
